# Mahjong Community: A Case Study on A Spontaneously Formed Adult Day Care Model in Underdeveloped Regions of China

**DOI:** 10.1093/geroni/igaf122.3808

**Published:** 2025-12-31

**Authors:** Yan Ming, Jie Yang, Yuehua Li

**Affiliations:** Capital Normal University, Beijing, Beijing, China (People’s Republic); East Carolina University, Greenville, North Carolina, United States; Minzu University of China, Beijing, Beijing, China (People’s Republic)

## Abstract

The underdeveloped areas in southwestern China are characterized by a triple pressure of low economic development, a high density of older adults, and a lack of long-term care (LTC) resources. Social isolation and loneliness are risk factors for mortality of older adults and negatively impact their health and wellbeing. A new model of home-based community care for older adults has emerged spontaneously, playing a unique role in filling the structural gap in the LTC system. We refer to it as the “Mahjong Community.” Through a case study, this article explores the formation and characteristics of this new LTC model and discusses its implications for LTC policies in China. Relying on neighborhood social capital and the mahjong culture, Mahjong Communities transform family space into adult day care settings during the daytime. An average of 15-20 older adults participated daily from 11:30 am to 3:30 pm. The host also accommodated older adults with disabilities who particularly risked social isolation. The activities included mahjong games, socialization, tea and snacks, meals and gift exchanges during holidays. The participants and host formed an emotional bond which created a sense of community. The daily cost of individuals was four yuan (less than one dollar). This low-cost model creates sustainable micro-economic units across the studied geographic area and proves to be scalable across the country due to the similar mahjong culture in different regions. The government should provide support in areas such as improving universal design in infrastructure and subsidies to scale these communities.

